# The application of ChatGPT in nursing: a bibliometric and visualized analysis

**DOI:** 10.3389/fmed.2024.1521712

**Published:** 2024-12-18

**Authors:** Peng Wang, Qian Zhang, Wenyu Zhang, Jing Sun

**Affiliations:** ^1^The International Medical Services, Peking Union Medical College Hospital, Peking, China; ^2^The Neonatal Intensive Care Unit, Peking Union Medical College Hospital, Peking, China; ^3^School of Nursing, Dalian University, Dalian, Liaoning, China; ^4^School of Nursing, Peking University, Peking, China

**Keywords:** ChatGPT, nursing, knowledge hotspots, visualized analysis, CiteSpace

## Abstract

**Objective:**

With the development of ChatGPT, the number of studies within the nursing field has increased. The sophisticated language capabilities of ChatGPT, coupled with its exceptional precision, offer significant support within the nursing field, which includes clinical nursing, nursing education, and the clinical decision-making process. Preliminary findings suggest positive outcomes, underscoring its potential as a valuable resource for enhancing clinical care. However, a comprehensive analysis of this domain is lacking, and the application of bibliometric methods remains rare. This study aims to describe and predict the developmental trajectory of the discipline, identify research hotspots and trends, and provide a comprehensive framework for the integration of ChatGPT in nursing.

**Methods:**

Following the development of a search strategy in collaboration with librarians, the implementation of this strategy occurred in the Web of Science Core Collection (WoSCC) on June 30, 2024. For bibliometric and visual analyses—including evaluations of sources, institutions, countries, author collaboration networks, and keywords—Bibliometrix (version 4.4.2) and CiteSpace (version 6.2.R2 Basic) were employed.

**Results:**

A total of 81 articles published by 67 authors were retrieved from the Web of Science Core Collection database, covering the period of June 30, 2024. The number of published studies has exhibited an increasing trend. The “European Journal of Cardiovascular Nursing” emerged as the most productive journals, while the USA, the UK, and China were identified as the leading countries in terms of publication output. The top 10 keywords identified in this study include artificial intelligence, nursing education, large language models, ChatGPT, natural language processing, generative artificial intelligence, care, nursing practice, clinical decision-making, and deep learning.

**Conclusion:**

ChatGPT is an emerging tool in the nursing field, currently in the foundational research phase. While there is significant international collaboration, cooperation among author groups remains somewhat limited. Studies focusing on ChatGPT in nursing primarily concentrate on two key themes: (1) the deep learning of ChatGPT in nursing and (2) the feasibility of its application. It is essential for nurses across various specialties to collaborate in exploring the diverse applications of ChatGPT within their domains, thereby fostering the ongoing development and enhancement of this technology.

## Introduction

On November 30, 2022, OpenAI launched ChatGPT, a text-based chatbot powered by a large language model (1). As ChatGPT continues to evolve, its significance and application within the healthcare industry are becoming increasingly apparent (2). The advanced language capabilities of ChatGPT, combined with its impressive accuracy, offer essential support in nursing (3), which includes domains such as clinical nursing (4–6), nursing education (6–10), and clinical decision-making (11, 12). Preliminary findings have shown promising results, suggesting its potential as a tool for clinical care assistance (1, 13). ChatGPT could transform the nursing profession and positively impact the health of both patients and healthcare providers (9).

Despite the increasing interest in this technology, significant knowledge gaps remain regarding its usage patterns in nursing, particularly concerning its advantages and potential drawbacks (14). Issues such as misinformation (8), digital dependence (15), and ethical dilemmas (16, 17) have also been raised by nursing professionals. Despite the increasing body of research in this area, there remains a lack of comprehensive analysis within the nursing field, and the application of bibliometric methods in this domain is still relatively uncommon. This research contributes to the nursing literature by providing a detailed examination of ChatGPT's role in nursing, a topic that has not been adequately explored.

This study aims to demonstrate, evaluate, and predict the developmental trajectory of nursing's evolution and advancement influenced by the integration of ChatGPT. It seeks to explore new roles, applications, and potential future directions, while also identifying existing hotspots and trends in the utilization of ChatGPT within the nursing discipline. Additionally, the study endeavors to establish a comprehensive framework that addresses the various applications and implications of ChatGPT in the nursing sector.

## Methods

This bibliometric and visual analysis was conducted via the R bibliometric package and CiteSpace to examine publications concerning the use of ChatGPT in nursing research.

### Search strategy

To ensure a high level of quality and a stringent selection process for the literature, we collaborated with a librarian to develop our search strategy (18), which we executed within the Web of Science Core Collection (WoSCC). Recognized globally as one of the oldest and most reputable sources of research publications and citations, the WoSCC database provides comprehensive and reliable information (19). It is widely regarded as the primary database utilized for bibliometric studies (20). Given the interdisciplinary applications of ChatGPT in nursing, the extensive coverage offered by WoSCC enables us to effectively gather relevant literature (21). The search strategy was formulated as follows: TS = (“ChatGPT” OR “Chat-GPT” OR “Chat GPT” OR “GPT-3.5” OR “GPT-4”) and TS = (“nurs^*^” OR “care”) from the Web of Science Core Collection. The search was executed on June 30, 2024, and focused on publications related to ChatGPT in nursing research, which served as the inclusion criterion. The criteria established for the inclusion of studies in this research were as follows: (1) only articles published in English, and (2) research relevant to the domain of generative artificial intelligence in nursing. No exclusion criteria were defined for this investigation. The literature screening was conducted independently by the first and second authors, who began by reviewing the titles and abstracts of each paper according to the predetermined inclusion standards to identify works requiring full-text evaluation. The final phase of the screening process involved a comprehensive review of the complete texts to ensure compliance with all established criteria. Any disagreements that arose during the literature review were resolved through group discussions. The search process yielded 99 studies from the database. After assessing for duplicate publications and applying the inclusion criteria, a total of 81 publications were selected for bibliometric and visual analysis.

### Bibliometric analysis methodology

We utilized the Biblioshiny web interface within RStudio, along with the bibliometric package, to perform the bibliometric analysis (22, 23). For the data analysis in this study, we employed Bibliometrix version 4.1.4 software. Following the installation of the Bibliometrix R package, the Bibliometrix web interface was launched via the command “bibliometrix::biblioshiny().” We analyzed influential factors, including sources, articles, authors, affiliations, institutions, and countries, that significantly impacted the application of ChatGPT in nursing research within the selected timeframe.

### Visualized analysis methodology

CiteSpace was utilized to conduct a visual analysis. This free Java application, which is based on network analysis and visualization (24), is specifically designed to address inquiries regarding the field of knowledge, a concept that broadly encompasses scientific fields, research domains, or scientific disciplines (25). For data processing, the selected timeframe spans from 2023 to 2024, with a time slice of 1 year. All relevant items, such as titles, abstracts, supplementary keywords (ID), author keywords (DE), and various other identifiers for nodes, were included, while default values were applied to the remaining items. The critical path method was employed to analyze data collection elements, construct a knowledge map, utilize co-occurrence maps to investigate research hotspots over the years, and apply time-zone views to elucidate the developmental relationships among these research hotspots.

## Results

### Publication characteristics

**Figure 1:**
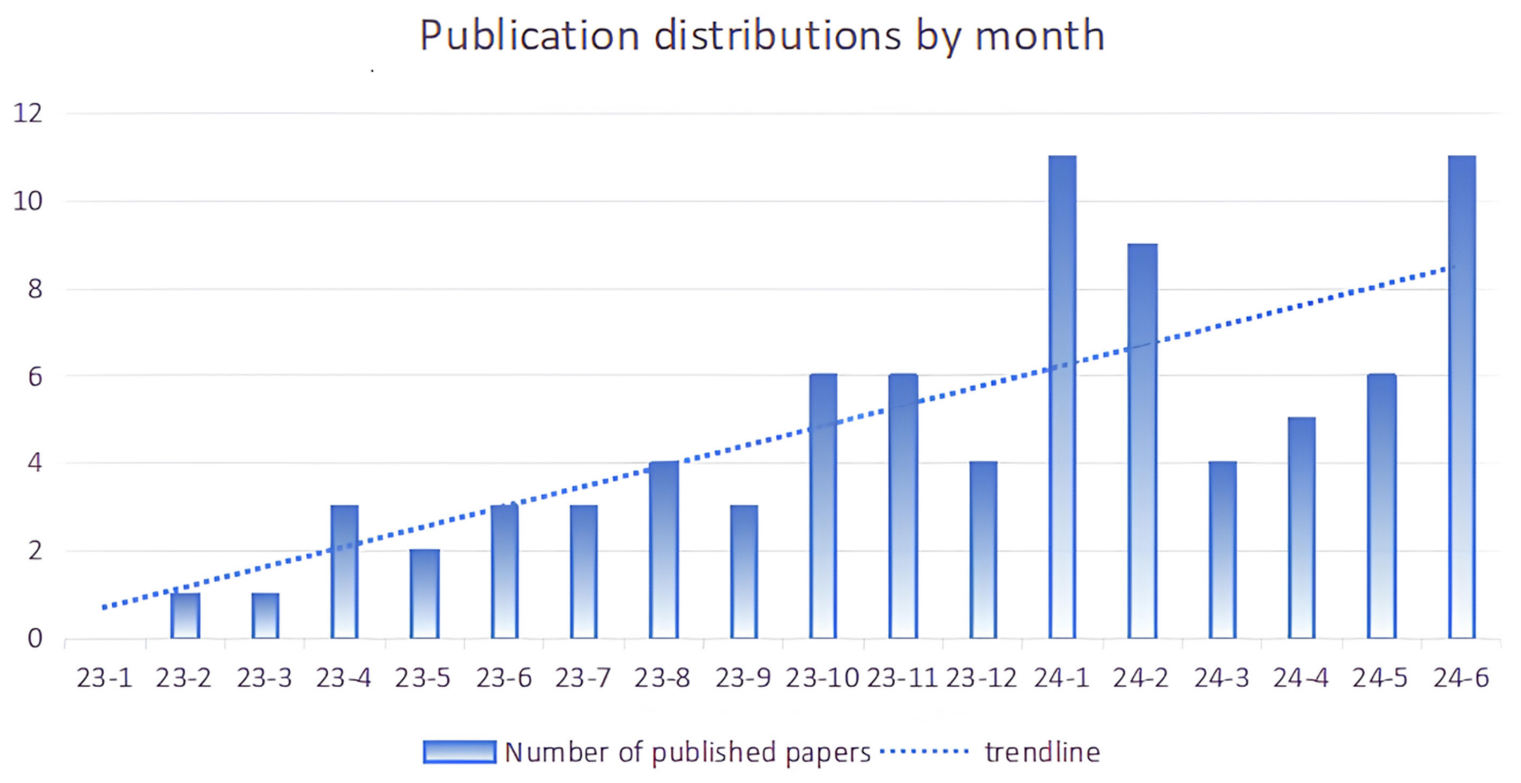
Number of articles published per month.

Since the release of ChatGPT in November 2022, the publication distributions by month, as depicted in Figure 1, encompassed publications from December 2022 to June 2024. A total of 81 publications were included in the analysis, comprising 46 articles, 16 editorial materials, nine letters, eight reviews, and two proceedings. The growth rate of published studies has exhibited an increasing trend, as indicated in Figure 1. The number of papers published in 2023 (*n* = 35) was lower than that published in the first half of 2024 (*n* = 46).

### Analysis of sources

**Figure 2:**
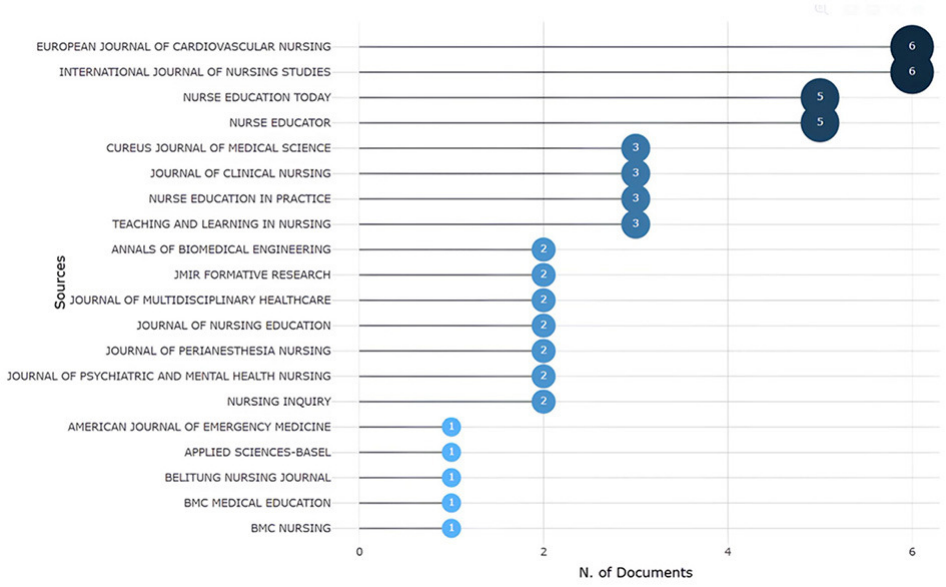
The top 20 most published sources.

The source analysis involved identifying the most relevant sources, applying Bradford's law, and examining the local impact of these sources. The results revealed the top 20 most relevant sources that have published works related to ChatGPT in nursing. The “European Journal of Cardiovascular Nursing” and the “International Journal of Nursing Studies” ranked highest, each producing six documents. They were followed by “Nurse Education Today” and “Nurse Educator,” which each published five works, along with the others detailed in Figure 2.

**Figure 3:**
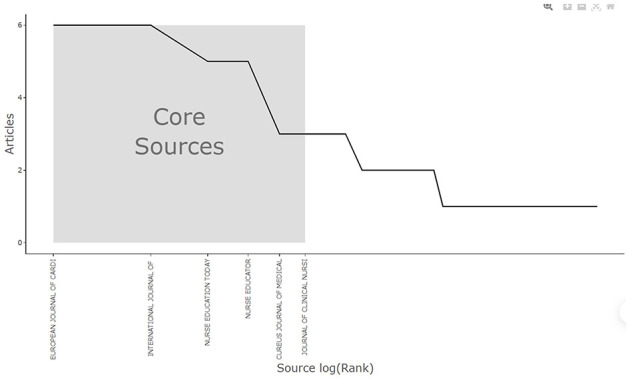
Core sources by Bradford's Law.

Bradford's Law suggests that the most significant sources can be identified among the first 50 articles (26). It categorizes sources into different zones. The first zone is considered the core source, encompassing the majority of relevant articles from the initial 50 selected. Among the top 20 sources, the “European Journal of Cardiovascular Nursing” and the “Journal of Clinical Nursing” are classified in Zone 1, indicating that these are the primary sources for relevant searches (see Figure 3 and Table 1).

**Table 1:** A list of core sources by Bradford's law.

**Source**	**Rank**	**Freq**	**cumFreq**	**Zone**
European Journal of Cardiovascular Nursing	1	6	6	Zone 1
International Journal of Nursing Studies	2	6	12	Zone 1
Nurse Education Today	3	5	17	Zone 1
Nurse Educator	4	5	22	Zone 1
Cureus Journal of Medical Science	5	3	25	Zone 1
Journal of Clinical Nursing	6	3	28	Zone 1
Nurse Education in Practice	7	3	31	Zone 2
Teaching and Learning in Nursing	8	3	34	Zone 2
Annals of Biomedical Engineering	9	2	36	Zone 2
JMIR Formative Research	10	2	38	Zone 2
Journal of Multidisciplinary Healthcare	11	2	40	Zone 2
Journal of Nursing Education	12	2	42	Zone 2
Journal of Perianesthesia Nursing	13	2	44	Zone 2
Journal of Psychiatric and Mental Health Nursing	14	2	46	Zone 2
Nursing Inquiry	15	2	48	Zone 2
American Journal of Emergency Medicine	16	1	49	Zone 2
Applied Sciences-Basel	17	1	50	Zone 2
Belitung Nursing Journal	18	1	51	Zone 2
BMC Nursing	20	1	53	Zone 2
Clinical Simulation in Nursing	21	1	54	Zone 2
Diagnostics	22	1	55	Zone 2

An analysis of the impact of sources, which is based on the weighting of their h-index, g-index, and m-index (27, 28), indicated that the journals with the highest impact are “Nurse Education Today,” “European Journal of Cardiovascular Nursing,” and “International Journal of Nursing Studies,” as evidenced by their respective h-index, g-index, and m-index (see Table 2). Notably, the two journals with the highest citation counts are “Nurse Education Today” and “European Journal of Cardiovascular Nursing.” The majority of journals with over 30 total citations are related to the field of education.

**Table 2:** A list of source local impact.

**Source**	**h_index**	**g_index**	**m_index**	**TC**
Nurse Education Today	5	5	2.5	94
European Journal of Cardiovascular Nursing	4	6	2	94
International Journal of Nursing Studies	3	3	1.5	13
Annals of Biomedical Engineering	2	2	1	28
Journal of Clinical Nursing	2	3	1	33
Journal of Nursing Education	2	2	1	7
Nurse Education in Practice	2	3	1	40
Nurse Educator	2	5	1	70
Teaching and Learning in Nursing	2	2	1	7
American Journal of Emergency Medicine	1	1	1	1
Applied Sciences-Basel	1	1	0.5	5
Belitung Nursing Journal	1	1	0.5	24
BMC Medical Education	1	1	1	3
Clinical Simulation in Nursing	1	1	1	1
Cureus Journal of Medical Science	1	3	0.5	12
Diagnostics	1	1	1	10
Educational Technology and Society	1	1	1	6
Electronics	1	1	0.5	17
Family Medicine and Community Health	1	1	1	5

### Affiliation and country analysis

**Figure 4:**
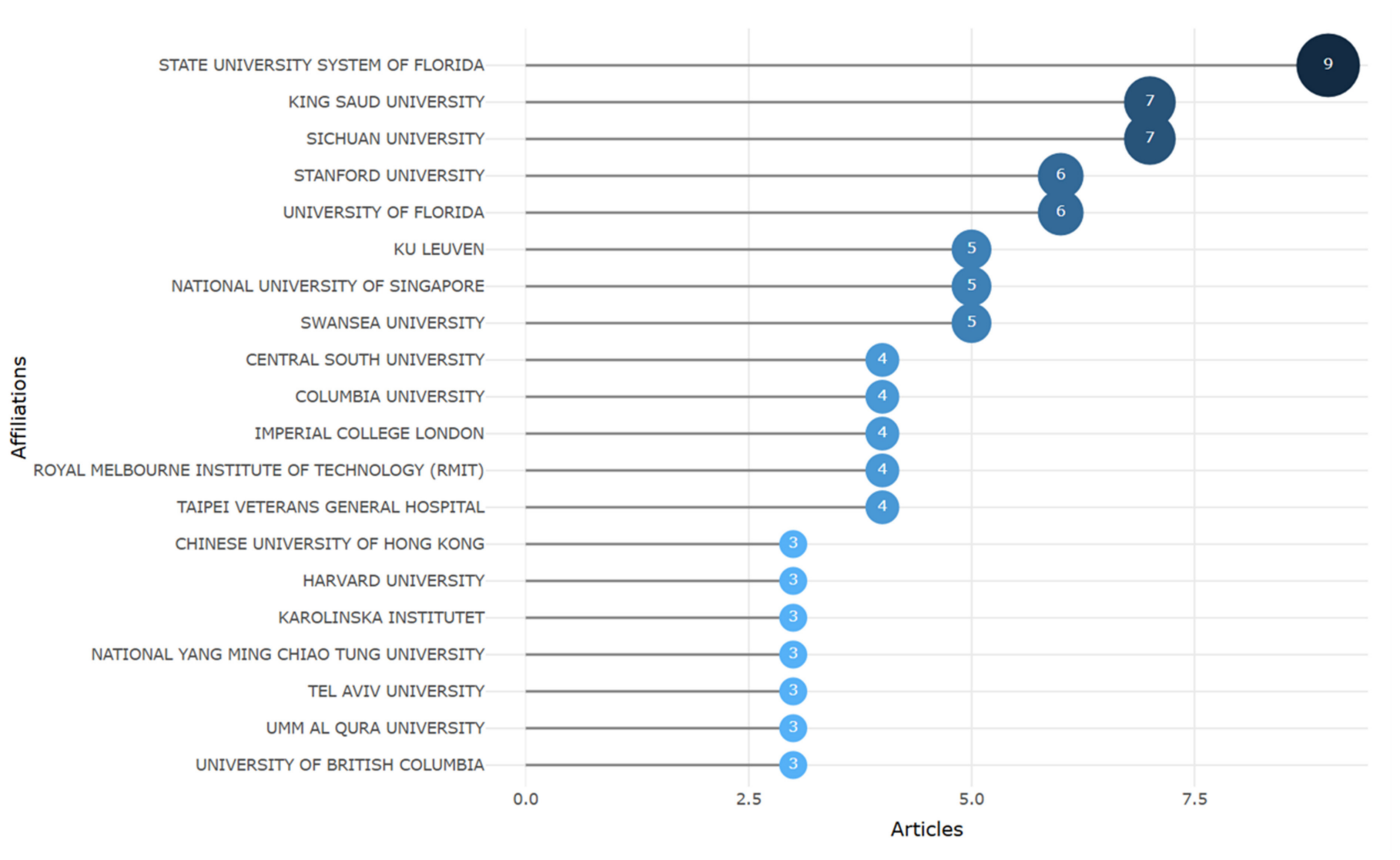
The top 20 most published affiliations.

We identified the top 20 most relevant affiliations, which represent the contributions of prominent institutions in producing articles on the selected topic. The State University System of Florida leads with nine articles, followed by King Saud University and Sichuan University, each with seven articles, along with the others mentioned in Figure 4. This underscores these institutions as key players in ChatGPT in nursing research.

**Figure 5:**
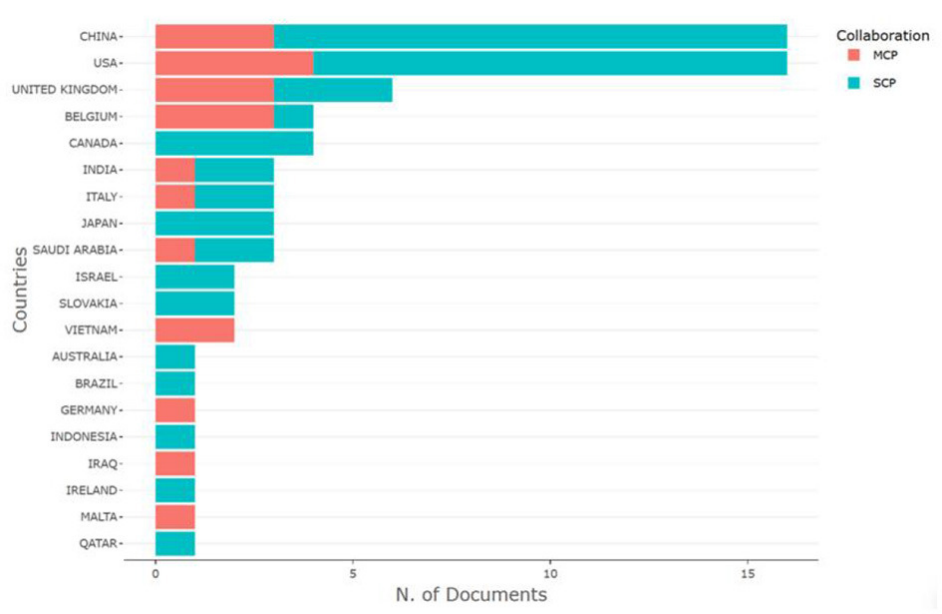
The top 20 most productive corresponding authors' countries.

**Figure 6:**
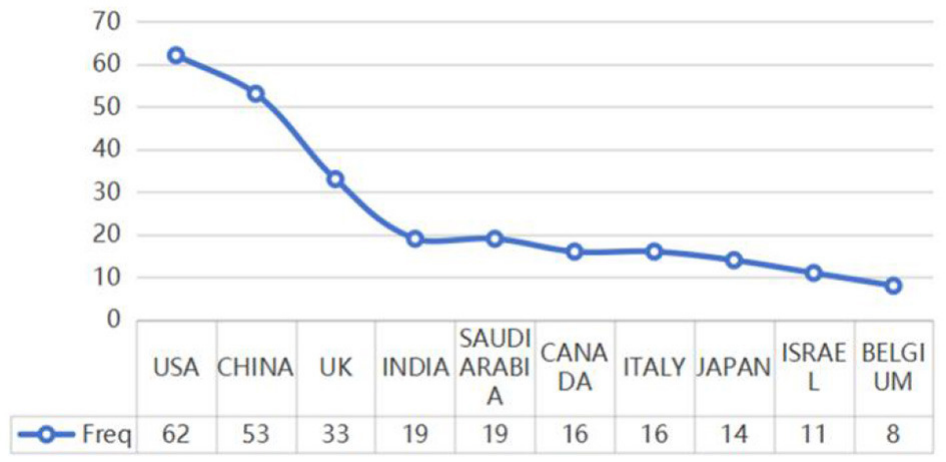
Top 10 most frequency of scientific production.

The top 20 countries with the most relevant corresponding authors have been identified on the basis of their simple publications (SCP) and multiple publications with other countries (MCP) (23). China (13 SCPs, 3 MCPs) and the USA (12 SCPs, 4 MCPs) led, with a total of 16 articles each. Additional countries are detailed in Figure 5. The global scientific contributions of these top 20 countries have been assessed, with the USA at the forefront, exhibiting a frequency of scientific production of 62, followed by China with 53, and the UK with 33 (see Figure 6). These statistics highlight the dominant roles of the USA and China in the research surrounding ChatGPT in nursing.

**Figure 7:**
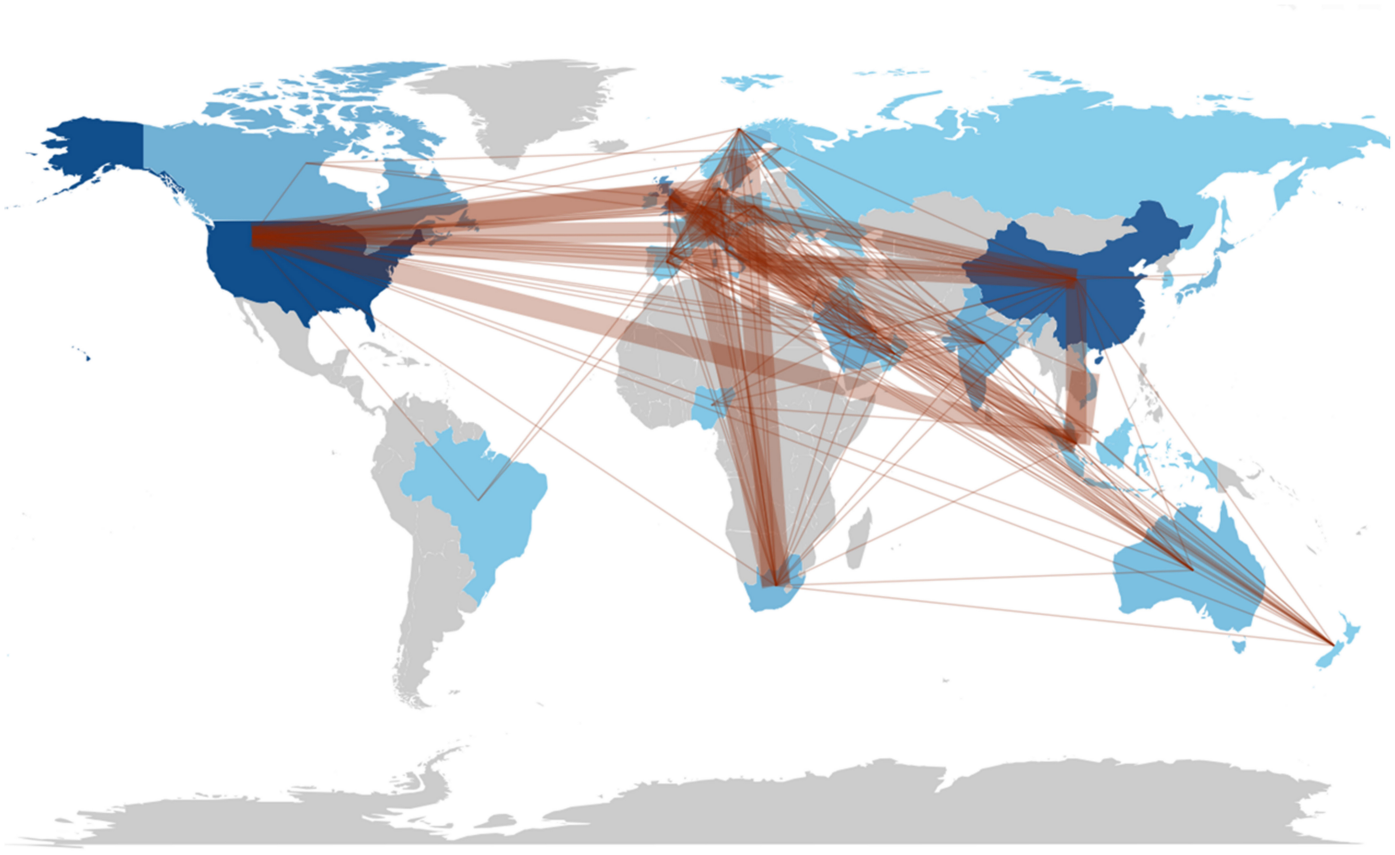
Collaboration world map.

The collaboration world map illustrates the affiliations of authors based on their countries (Figure 7). The results indicate that the continents exhibit varying levels of strong collaboration. Upon evaluating the connections between countries, we find that the USA leads with 32 links, closely followed by the United Kingdom with 33 links and China with 24 links. The USA, recognized as one of the most active countries, maintains two or more partnerships with China, Denmark, Singapore, Switzerland, and the UK. The proximity of the nodes or circles on the map, along with the thickness of the connecting lines, suggests that the number of national publications is directly proportional to the degree of cooperative association.

### Author cooperation network

**Figure 8:**
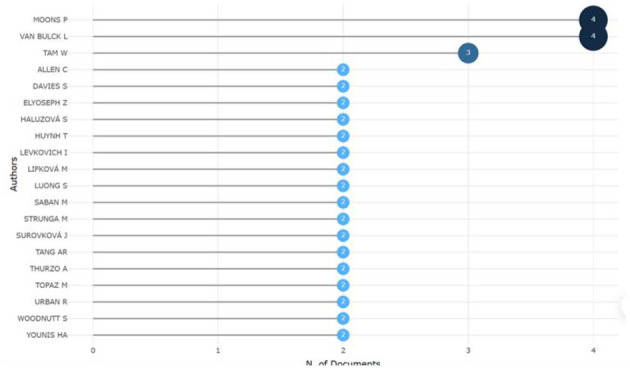
The top 20 most productive authors.

Through the analysis of the number of papers published by the authors and their cooperation network, we identified 67 authors who are engaged in the study of CiteSpace within nursing research. On the basis of the frequency measure of the number of documents authored, Moons and Van Bulck lead their peers, each having produced four articles. All other relevant authors are presented in Figure 8.

**Figure 9:**
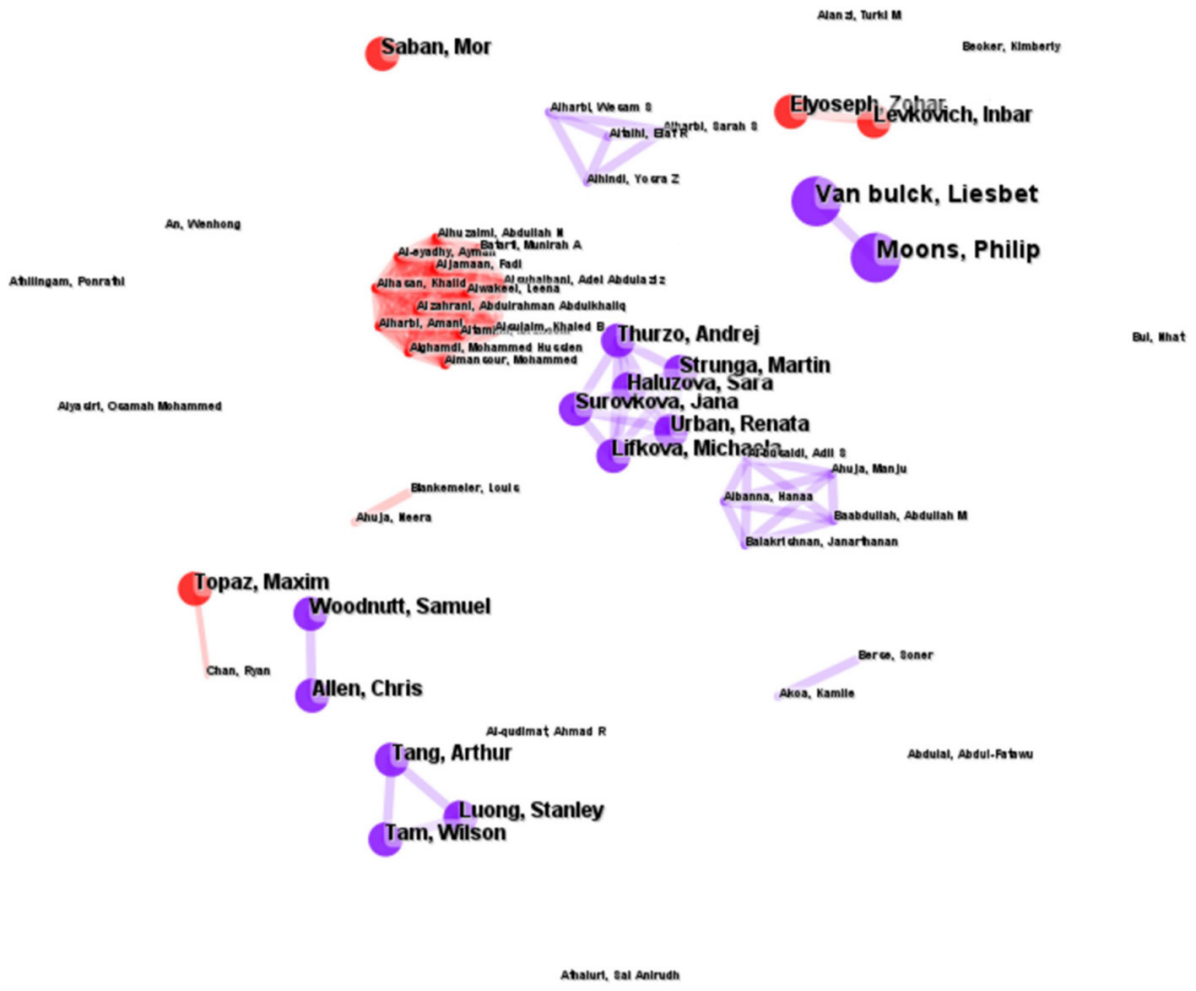
Author cooperative relationship.

By analyzing author cooperative relationships, we observed a decentralized distribution among scholars (Figure 9). The analysis encompasses the 67 most cited contributors and 150 co-citation links. Evidence of collaborative teams among scholars is apparent, as mutual interactions occur among team members; however, each team experiences weak external collaboration. This suggests that although the research topics are multidisciplinary, they are primarily studied independently by various teams across different disciplines.

### Analysis of keywords

**Figure 10:**
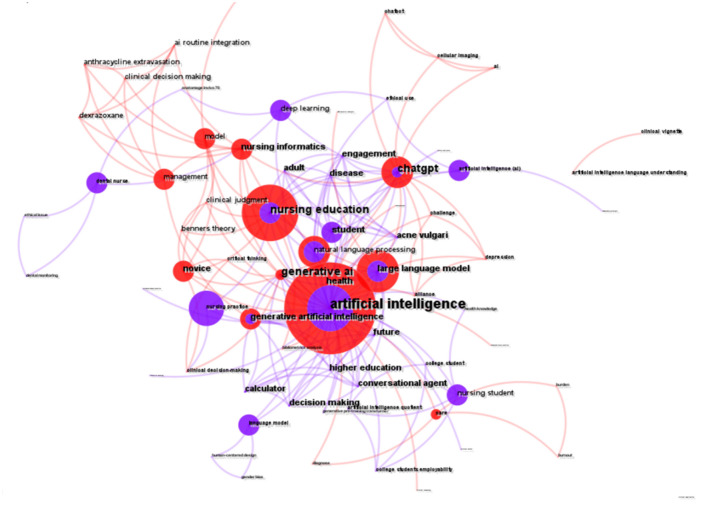
Co-occurrence network of keywords.

To explore research hotspots and cutting-edge topics, we analyzed the co-occurrence network of keywords. As illustrated in Figure 10, the connecting lines between various keywords are intricate, indicating complex interconnections. The top 10 keywords include artificial intelligence, nursing education, large language model, ChatGPT, natural language processing, generative artificial intelligence, care, nursing practice, clinical decision-making, and deep learning. In the figure, “artificial intelligence” and “nursing education” are represented by larger nodes, signifying their substantial presence in the topic.

**Figure 11:**
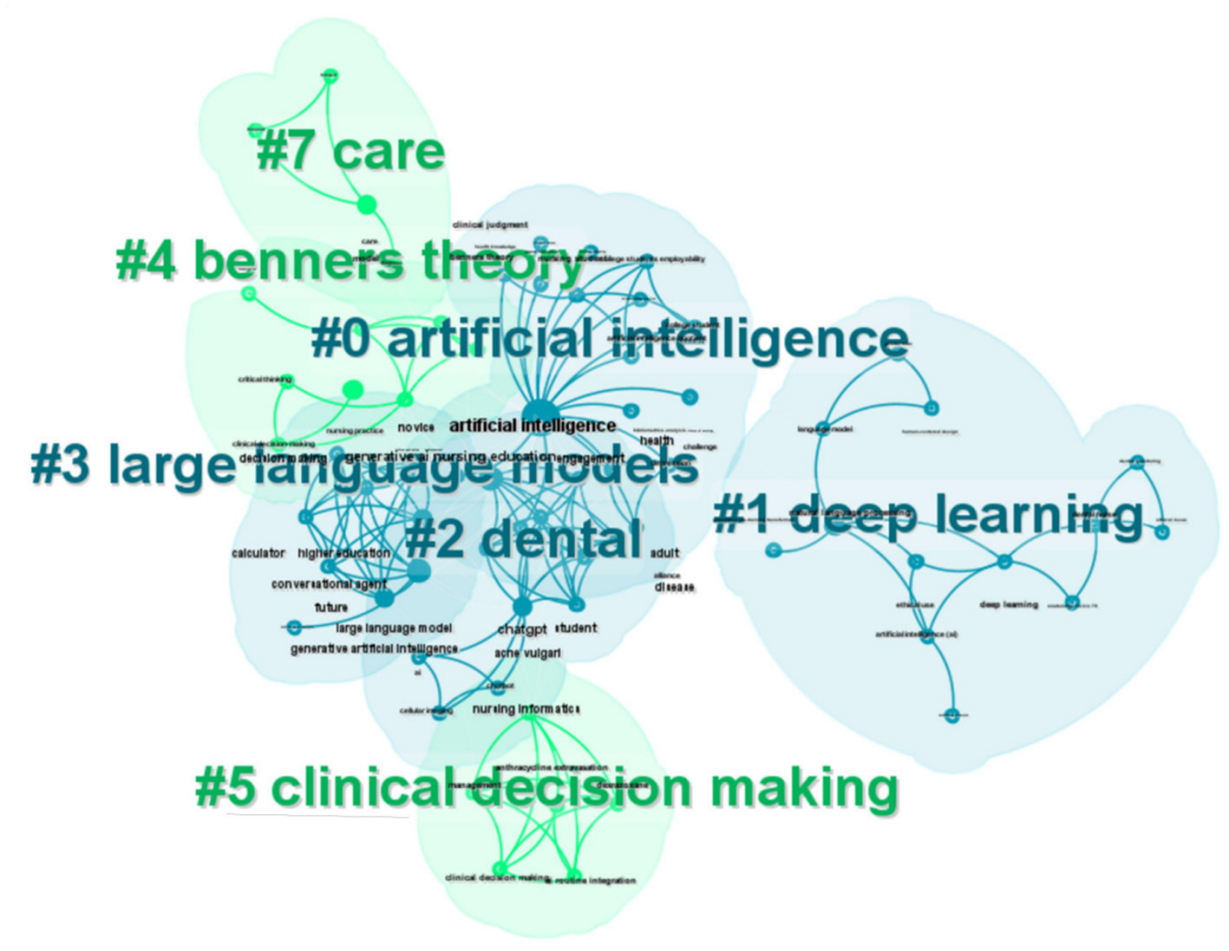
Clustering network of keywords.

The hierarchical arrangement of articles is organized through a clustering network (Figure 11). Co-occurring keywords are categorized into seven subclusters: #0 artificial intelligence, #1 deep learning, #2 dental, #3 large language models, #4 Benner's theory, #5 clinical decision making, and #6 care. The center node in Figure 8 represents the highest occurrence of the term “artificial intelligence” within the co-occurrence network. Key intermediaries such as “generative AI,” “nursing education,” and “decision making” serve to connect the clusters. The silhouette value for each cluster exceeded 0.8, indicating that the results are both reliable and significant (Table 3).

**Table 3:** A list of the clustering network.

**Cluster ID**	**Size**	**Silhouette**	**Year**	**Cluster label**	**Top terms (LSI)**
#0	17	0.846	2023	Artificial intelligence	Artificial intelligence; the-art language processing; nursing students; college student; nursing education; nursing education research; health knowledge; social responsibility; nursing informatics
#1	13	0.966	2024	Deep learning	Deep learning; image processing; machine learning; dental nurse; image segmentation; gender bias; machine translation; language models; human-centered design
#2	11	0.976	2023	Dental	Nursing education; artificial intelligence; narrative review; pedagogical approach; student assessment; proximal development; vygotskys zone; skin symptoms
#3	9	0.976	2023	Large language models	Large language models; generative artificial intelligence; conversational agent; nursing informatics; bibliometric analysis; scoping review
#4	8	0.816	2023	Benners theory	Skill acquisition; nursing education; benners theory; artificial intelligence; clinical decision-making
#5	6	0.954	2023	Clinical decision making	LLMS feasibility; AI routine integration; methodology; clinical decision making; healthcare innovation; nursing informatics; safety; multidisciplinary approach; multi-parametric analysis
#7	4	0.973	2023	Care	Burnout; burden; care; nurse

**Figure 12:**
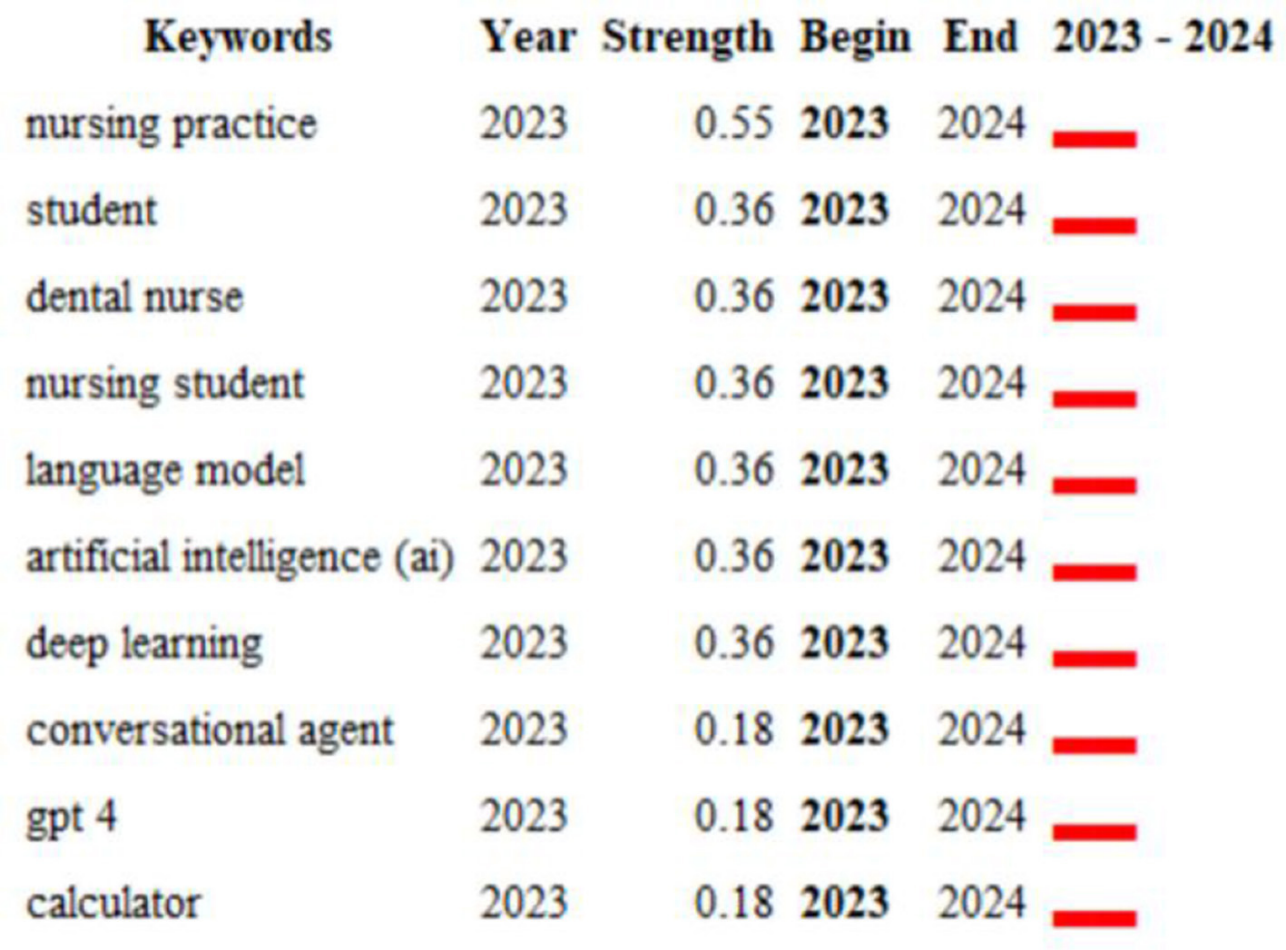
The top 10 keywords with the strongest citation bursts.

The term “burst vocabulary” refers to a set of words that are frequently cited over a specific period (Figure 12). The top 10 keywords associated with this duration include nursing practice (0.55), student (0.36), dental nurse (0.36), nursing student (0.36), language model (0.36), artificial intelligence (AI) (0.36), deep learning (0.36), conversational agent (0.18), GPT-4 (0.18), and calculator (0.18). These keywords indicate a significant increase in scholarly attention to various aspects of ChatGPT in nursing, highlighting the research trends within this domain. It is evident that disciplines such as nursing practices, students, dental nurses, and nursing students are increasingly focused on the application of new technologies, demonstrating heightened sensitivity and innovation in response to advancements in science and technology.

## Discussion

Hobensack et al. suggested that nurses across various domains—such as practice, research, education, and policy—are expected to be influenced by the use and application of large language models, with nearly all (93%) of the reviewed articles identifying ChatGPT as a prominent example (29). Although there are some limitations in this article, it effectively underscores the significance of ChatGPT within the nursing field. Furthermore, bibliometric trends suggest that this field is actively evolving and characterized by early exploration and significant growth. This gradual increase reflects increasing interest, likely driven by advancements in ChatGPT and a growing awareness of its potential applications within the nursing profession (30). The dynamic nature of this field emphasizes the potential for further advancements and discoveries, indicating that we are still in the process of comprehensively understanding its full impact and possibilities (31).

The findings of the most relevant sources indicate that similar results are achieved when sources are analyzed from different perspectives. The leading sources encompass a variety of topics, including nursing education (32), cardiovascular care (33), emergency care (34), perianesthesia nursing (35), psychiatric health (36), and family and community care (37, 38), thereby highlighting the extensive applicability of the ChatGPT within the nursing profession. Nine of the top 20 journals focus on education, reflecting ChatGPT's current areas of emphasis in conjunction with nursing. Among these educational articles, the majority conclude that ChatGPT is feasible for nursing education; however, they also acknowledge limitations and ethical dilemmas that could inform updates to the ChatGPT version (7, 9, 39). In our bibliometric study, we employed Bradford's Law to categorize the sources into distinct zones, which aids in identifying the principal journals within a specific subject area (40). White (66) noted that Bradford's Law could lead to the misconception that articles published in the primary journals of a field are generally of higher quality than those distributed across peripheral journals (41). To mitigate the inaccuracies arising from this bias, we concurrently assessed the h-index, g-index, and m-index. The results indicated that sources in Zone 1 exhibited a significant impact, thereby enabling us to further identify high-quality sources within the domain of ChatGPT in nursing.

The literature on the measurement of affiliation and country indicates that the high volume of articles not only reflects a strong institutional emphasis on this area of research but also suggests access to essential resources, such as funding, talent, and data, which are crucial for sustained academic productivity (42, 43). The presence of institutions from the United States, China, and Saudi Arabia further underscores that the exploration of ChatGPT in nursing research is a global phenomenon characterized by geographical diversification. These institutions possess robust interdisciplinary collaborations that integrate expertise from both nursing and computer science, fostering innovation and the exchange of ideas (43). The SCP highlights the strong national research capabilities and initiatives of the USA and China in this interdisciplinary field. Additionally, the MCP highlights the role of these two countries in international collaboration, facilitating the global exchange of knowledge and expertise in this domain (38). For example, China and the United States have collaborated on a multidisciplinary approach to address the opportunities and challenges posed by artificial intelligence (44), as well as the applications of ChatGPT in nursing education (45). Academic collaboration among various countries or regions can significantly enhance the dissemination of knowledge and foster academic exchange (46). Although ChatGPT is an emerging technology, collaboration among nations across all continents underscores the globalization and significance of ChatGPT research in the field of nursing. Advancements in technology and the deepening of research efforts suggest that such cooperation will become increasingly essential in the future.

The development of large AI models necessitates closer and more intense collaboration among domain experts, as well as the gradual establishment of regulations (47). In the author collaboration network analyzed by Citespace, the collaboration density is measured at 0.0678, indicating that the authors' cooperative efforts are dispersed (48), which may stem from differing valuations of the subject matter by each team. The group comprising Moons and Van Bulck primarily focuses on the trustworthiness and value of ChatGPT (6, 49). Tam et al.'s group focused on nursing education in the age of artificial intelligence (7), whereas Allen group collaborated on mental health (50). This suggests that, despite the multidisciplinary nature of the research topics, they are predominantly investigated independently by various teams across different disciplines. It is plausible that the extensive scope of nursing as a subject area has prompted these teams to explore specific nursing specialties in divergent directions. Although there appears to be limited collaboration among the teams, this does not necessarily imply a deficiency of teamwork in the research concerning ChatGPT in nursing. As research on ChatGPT intensifies and the volume of studies within the same specialty increases, the focus may gradually shift from assessing the feasibility of ChatGPT in nursing to deep learning itself. Consequently, the trend of collaboration among different teams may increase in the future.

The analysis of hotspot evolution revealed that ChatGPT has been extensively studied within the realms of nursing education, clinical decision-making, and management, highlighting its significant application in the nursing field. As an emerging artificial intelligence technology, ChatGPT has spurred advancements in both nursing education and clinical decision-making (51, 52). The interconnectedness of nursing and ChatGPT is evident, as both domains appear to support each other's progression. By utilizing the keyword clustering knowledge graph and collinear network clustering table, it becomes clear that most clusters exhibit overlap. Among the seven identified clusters, the clusters pertaining to artificial intelligence, dental, large language models, and Benner's theory are closely interconnected, whereas the clusters related to deep learning, clinical decision-making, and care are more peripheral due to their looser connections. This observation indicates that current research is still in the early stages of foundational data research and technological development. ChatGPT is still in its preliminary stages, and the theoretical foundations and data models of the four interconnected clusters are expected to maintain a dominant position in future research. The dental cluster is closely linked to other clusters, primarily emphasizing nursing education. This alignment indicates that nursing education is consistent with current research hotspots and focal points. Additionally, topics such as deep learning, clinical decision-making, and patient care reflect the continuous emergence of new areas of inquiry. ChatGPT is anticipated to engage in more comprehensive collaborative research grounded in the theoretical frameworks of clinical decision-making and patient care. Currently, the application of ChatGPT in nursing primarily revolves around nursing education, clinical decision-making, clinical nursing practice, automated writing, and addressing common nursing inquiries. In the realm of nursing education, ChatGPT applications include vocational examinations, application attitude surveys, educational practices, and teaching design, among others. ChatGPT as a representative product, its application and research results also show the main advantages of “Anthropic Claude,” “Google Gemini” and other generative AI in the field of nursing, as well as their usability and research prospects. The results and discussion indicate that ChatGPT offers significant advantages in the nursing field, including user-friendliness, rapid response capabilities, data-driven content generation, and enhanced efficiency. A representative example is the applications and research findings related to ChatGPT, which also emphasize the relevance of other generative AI models, such as “Anthropic Claude” and “Google Gemini,” within the nursing domain, thereby highlighting their usability and research potential.

Nearly all the articles evaluated the risks associated with the ChatGPT. Perspectives on this issue vary; some scholars adopt a negative stance, indicating that further research is necessary (53–55), whereas the majority advocate embracing the challenge and seizing the opportunities presented (13, 39, 56). Ethical considerations are a crucial element that must not be overlooked. Issues related to reliance on technology (57), misdiagnosis and treatment errors (58), data security breaches (59), and the trustworthiness of patients (60) must be addressed when utilizing ChatGPT. Future studies should continue to examine the ethical ramifications of artificial intelligence concerning patient confidentiality and data protection (2), the accuracy and credibility of information (61), autonomy in decision-making (62), and transparency (63), to enhance the integration of ChatGPT within the nursing field.

We acknowledge the limitations of our study. (i) CiteSpace's dependence on specific data sources is primarily evident in its connection to particular databases, notably the Web of Science (WoS) and others. This dependence constrains the scope and comprehensiveness of CiteSpace's data collection and analysis, potentially omitting relevant literature that is not included in these databases (21, 64). Our investigation is confined to publications included in the Web of Science Core Collection (WoSCC), which does not encompass all journals; this may lead to the oversight of articles in other databases, such as Scopus and PubMed. Nevertheless, the WoSCC is a comprehensive and well-organized database that is extensively utilized across various scientific disciplines, and the quality of papers within this source is widely recognized and employed in most scientometric studies. (ii) While CiteSpace is capable of identifying significant patterns and trends within scientific literature, it does not function at its full potential for conducting in-depth analyses of specific fields or subjects (65). Therefore, it may be necessary to employ additional tools or techniques to gain more comprehensive insights. To complement these limitations, the bibliometric package was applied to conduct more in-depth statistical analysis of the data, such as the top 20 most relevant affiliations, Bradford's Law, the impact of sources, SCP, MCP, etc. So as to dig out deeper academic information. (iii) Our analysis was limited to English-language articles published in reputable peer-reviewed scientific journals, which may introduce potential publication bias.

## Conclusions

ChatGPT is an emerging tool in the field of nursing and is currently in the basic research stage. To our knowledge, the present study represents the first bibliometric analysis of the application of the ChatGPT in nursing, identifying key contributors, including countries, authors, and journals. Our findings indicate that the United States and China are the leading countries in terms of publication volume and that international collaboration is robust. However, there is limited cooperation among author groups, which can be attributed to differences in specialties. Therefore, it is essential for nurses from various specialties to collaborate in exploring the diverse applications of ChatGPT within their fields, thereby facilitating the further development and enhancement of this technology. Our hotspot analysis revealed that publications on ChatGPT in nursing have focused on two main themes: (1) the deep learning of ChatGPT in nursing and (2) the feasibility of its application. In addition to discussing the use of ChatGPT in nursing, we provide several suggestions for academics to conduct empirical studies in this area: (1) The literature currently lacks randomized controlled trials and qualitative studies; thus, the effects of ChatGPT could be evaluated via a variety of research designs. (2) By integrating different artificial intelligence tools (such as DeepL, especially AI, and Resemble AI) and technologies (including virtual reality, augmented reality, and mobile applications) with ChatGPT, we can investigate the effects of these combinations on nursing practice. (3) The literature on the application of ChatGPT in nursing tends to be fragmented, particularly concerning foundational data studies. It is feasible to enhance the application of ChatGPT across various practice areas and identify commonalities through collaborative efforts. By addressing these research priorities, we can substantially advance our understanding of the potential of ChatGPT as a tool in nursing and develop a diverse range of strategies.
